# The Influence of Habitat Heterogeneity and Human Disturbance on Trophic Interactions of *Sebastiscus marmoratus* and *Scorpaenopsis cirrosa* in Wanshan Archipelago

**DOI:** 10.1002/ece3.71869

**Published:** 2025-07-24

**Authors:** Hongyu Xie, Yu Liu, Teng Wang, Peng Wu, Yayuan Xiao, Chunling Wang, Jian Zou, Yong Liu, Jinhui Sun, Jianzhong Shen, Xuefu Ao, Yanqiao Wang

**Affiliations:** ^1^ South China Sea Fisheries Research Institute, Chinese Academy of Fishery Sciences Key Laboratory of South China Sea Fishery Resources Exploitation & Utilization, Ministry of Agriculture and Rural Affairs/Observation and Research Field Station of Pearl River Estuary Ecosystem Guangzhou China; ^2^ Scientific Observation and Research Station of Xisha Island Reef Fishery Ecosystem of Hainan Province/Key Laboratory of Efficient Utilization and Processing of Marine Fishery Resources of Hainan Province/Sanya Tropical Fisheries Research Institute Sanya China; ^3^ College of Fisheries Huazhong Agricultural University Wuhan China; ^4^ Institute of Hydrobiology Chinese Academy of Sciences Wuhan China; ^5^ Collegeof Fisheries, Tianjin Agricultural University Tianjin China

**Keywords:** estuary, geographical variation, interspecific interactions, stable isotopes, trophic niche

## Abstract

Habitat heterogeneity and human disturbances drive variations in interspecific interactions among fish species. Trophic niche analysis is a crucial approach for understanding these interactions. This study investigates the interspecific interaction mechanisms of two scorpionfish species, *Sebastiscus marmoratus* and *Scorpaenopsis cirrosa* , across different habitat conditions in the Wanshan Archipelago of the Pearl River Estuary. Three island groups—Guishan Island, Wailingding Island, and Dongao & Wanshan Islands—were selected as study sites. Using carbon and nitrogen stable isotope analysis, we examined variations in their trophic relationships. The results revealed significant regional differences in the δ^13^C values of *S. marmoratus* and *S. cirrosa* (*p* < 0.05), indicating regional variation in their food sources. Bayesian mixing model analysis showed that at Guishan Island, annelids constituted the primary food source for both species. However, at Wailingding Island and Dongao & Wanshan Islands, their reliance on annelids significantly decreased, with a preference shift towards mollusks, reflecting region‐specific foraging strategies and niche differentiation. The δ^15^N values of the two species did not exhibit significant regional differences, suggesting that *S. marmoratus* and *S. cirrosa* occupy similar trophic levels across different regions. Trophic niche metrics, including CR, NR, CD, and TA, indicated that *S. cirrosa* has a broader trophic niche, suggesting a more flexible feeding strategy and a potential competitive advantage in resource utilization. The two species exhibited overlapping trophic niches, with distinct regional variations. Notably, at Guishan Island, where trophic niches were the broadest, potential competition between the species was most pronounced, which may be associated with higher human activity intensity. Differences in ecosystem structure among islands contributed to variations in food resource availability and niche space, ultimately shaping the foraging behaviors and adaptive strategies of *S. marmoratus* and *S. cirrosa* across regions. These findings provide theoretical insights into interspecific interactions in heterogeneous habitats and inform fisheries resource conservation.

## Graphical abstract

This study investigates how habitat heterogeneity and human activity shape trophic interactions between two scorpionfish species ( *Sebastiscus marmoratus* and *Scorpaenopsis cirrosa* ) in China's Wanshan Archipelago. Stable isotope analysis revealed regional shifts in their diets (e.g., annelids to mollusks) and broader niche use by *S. cirrosa* , with heightened competition in high‐human‐activity areas, underscoring the need for habitat‐specific conservation strategies. These findings highlight how ecosystem structure and anthropogenic pressures drive resource partitioning in marine species.

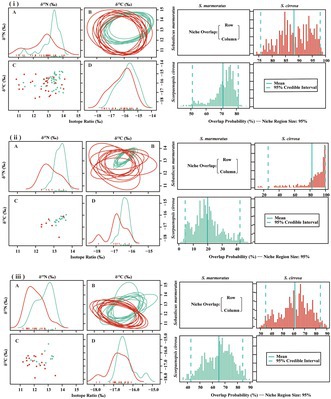

## 1 Introducation

The trophic niche is regarded as a key functional trait describing the role of consumers within an ecosystem. Both niche breadth and trophic position not only reveal an organism's role in the food web but also reflect its functional placement within the biological community (Hayden et al. 2019). Niche parameters can rapidly respond to changes in intraspecific and interspecific competition, as well as variations in prey abundance (Bearhop et al. 2004). Moreover, niche theory serves as a fundamental framework for explaining ecological processes that sustain biodiversity, species coexistence, and community stability. It provides essential insights into interspecific trophic interactions, coexistence and competition, food resource utilization patterns, and food web structures (Ke‐chang et al. 2009; Yu and Xu 2019). By comparing trophic relationships, researchers can uncover ecological linkages across different habitats, particularly in terms of trophic structure dynamics (Qu et al. 2021). Among various analytical approaches, stable isotope analysis has become a widely applied method for understanding various aspects of ecosystems and is one of the most powerful tools for studying marine trophic structures (Cherel and Hobson 2005). Carbon and nitrogen isotope ratios reflect an organism's long‐term feeding history (Peterson and Fry 1987), capturing trophic information accumulated throughout its life cycle and quantifying the primary food sources assimilated during growth (Mwijage et al. 2018). Additionally, statistical analyses of stable isotope data and isotope‐based models enable comparative assessments of trophic relationships, offering deeper insights into variations in trophic structures across different habitats (Jackson et al. 2011). For example, stable isotope analysis of various parrotfish species and functional groups in Zanzibar demonstrated a strong correlation between feeding variation and body size (Plass‐Johnson et al. 2012). Similarly, a stable isotope‐based study on the trophic ecology of *Dosidicus gigas* found that geographic differences were particularly evident in δ^15^N values, highlighting spatial variation in trophic position (Ruiz‐Cooley et al. 2010).

Growing evidence suggests that natural and anthropogenic disturbances have significantly altered the trophic structure of fish communities (Shin et al. 2005; Ullah et al. 2018; Nagelkerken et al. 2020). At the same time, habitat complexity within marine ecosystems is known to shape fish trophic niches (Christianen et al. 2017). Habitat fragmentation caused by human activities can disrupt ecological community structures and may lead to the loss of co‐evolved trophic interactions (Cross et al. 2013). A study on key fish species in the eastern Chukchi Sea, for example, revealed geographic variation in trophic niche space, with fish communities exhibiting different dietary sources across distinct environments (Marsh et al. 2017). As such, the relationship between trophic niche variation, environmental heterogeneity, and human disturbances has become a central theme in marine ecological research.

The Wanshan Archipelago is located in the northern South China Sea, within the Pearl River Estuary, positioned between Zhuhai and Hong Kong. It comprises more than 70 islands arranged in a semi‐circular pattern, forming a distinctive island chain structure (Tang et al. 2022). This unique geographical setting makes it an ideal location for studying the effects of habitat differentiation on fish communities. Additionally, the Wanshan Archipelago is situated within a subtropical monsoon climate zone and serves as a transitional area between terrestrial and marine ecosystems. This estuarine environment provides a habitat of significant ecological and economic importance.

In recent decades, intensified climate change and human disturbances have driven rapid transformations in marine and estuarine ecosystems (Roessig et al. 2005). These environmental changes have altered the structure and ecological functioning of estuarine biological communities, with fish assemblages and their food webs being particularly affected (Feyrer et al. 2015). Human‐driven factors such as overfishing and fisheries development directly impact fish distribution and food availability (Whitfield and Elliott 2002). Consequently, cumulative anthropogenic stressors have caused degradation of habitats and simplification of food web structures (Blaber et al. 2000). Notably, recent observations suggest that the ecological stability of the Wanshan Archipelago has been disrupted (Cross et al. 2013). The decline in fishery resources implies that marine habitats surrounding different islands may now exhibit distinct environmental profiles (Xu et al. 2022), ultimately driving changes in the trophic structure of marine food webs (Durante et al. 2022; Shi et al. 2024).

Given this context, understanding the trophic structure of ecologically important fish species in this archipelago is vital. *Sebastiscus marmoratus* and *Scorpaenopsis cirrosa* both belong to the family Scorpaenidae (order Scorpaeniformes), yet they are assigned to different genera: Sebastiscus and Scorpaenopsis, respectively. *Sebastiscus marmoratus* is a relatively small‐sized ovoviviparous species, with a maximum recorded length of 36.2 cm. It is widely distributed in the coastal waters of East Asia, including China, the Korean Peninsula, and the Japanese Islands south of Hokkaido. The species inhabits rocky reefs and seaweed beds, exhibits strong site fidelity and limited adult mobility, and its dispersal primarily occurs during the larval stage (Sun et al. 2022). Due to its palatable flesh and economic value, it is an important target species in regional demersal fisheries. *Scorpaenopsis cirrosa* is generally smaller, with a maximum length of approximately 23.1 cm, and is typically distributed in tropical and subtropical waters of the Indian Ocean and the western Pacific Ocean. It commonly inhabits shallow coral reefs and rocky substrates. Both species are benthic carnivores, primarily consuming small fishes, crustaceans, and mollusks (Harmelin‐Vivien et al. 1989; Xue et al. 2017). Despite their phylogenetic relatedness, their differences in geographic distribution and habitat preference suggest potential divergence in trophic strategies.

To investigate these differences, this study applies carbon and nitrogen stable isotope analysis to compare the trophic niche characteristics of *S. marmoratus* and *S. cirrosa* across multiple islands of the Wanshan Archipelago. Specifically, we evaluate their trophic niche breadth, overlap, and spatial differentiation to examine interspecific resource utilization and adaptive responses to habitat heterogeneity. The findings aim to clarify trophic interactions between these species and provide broader insights into how environmental variation and human disturbances influence marine food web structures.

## 2 Materials and Methods

### 2.1 Research Area

**FIGURE 1:**
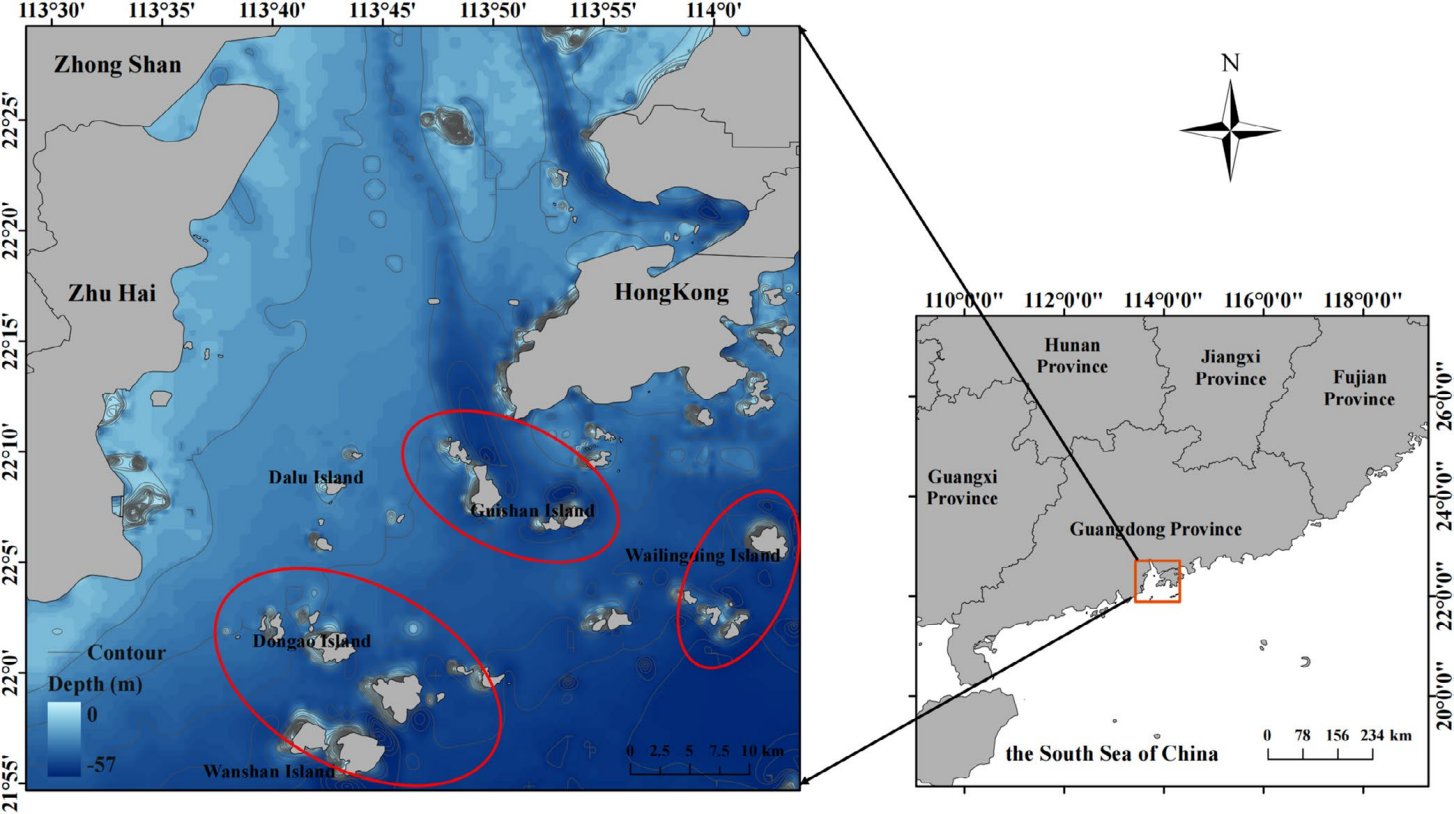
Sampling area of fish in Wanshan Islands.

The research was conducted in March and September 2021. Due to the proximity of Dong'ao Island to Wanshan Island, and considering the number of samples collected, the study area was divided into three groups: Guishan Island, Wailingding Island, and Dongao & Wanshan Island (Figure 1). The sampling range is entirely within the area marked by the red circles in Figure 1. Based on field records and bathymetric data, the water depths across sampling sites ranged from 4.5 to 25 m. A three‐layer gill net, with inner mesh sizes of 4 and 6 cm, was used to collect *S. marmoratus* , *S. cirrosa* , and other small benthic fishes, including shrimps and crabs. At each site, nets were deployed daily at approximately 16:00 and retrieved the following morning at around 7:00. This procedure was repeated for three consecutive days at each sampling location to ensure temporal consistency and adequate sample size. All captured specimens were immediately chilled and transported back to the laboratory, where they were stored at −20°C prior to processing.

### 2.2 Sample Collection and Treatment

A total of 68 *S. marmoratus* and 55 *S. cirrosa* specimens were collected for this study. Basic biological measurements, including body length and weight, were conducted in the laboratory. Body length was measured with an accuracy of 0.1 mm, while weight was measured with an accuracy of 0.01 g. According to previous studies, both *S. marmoratus* and *S. cirrosa* are carnivorous benthic feeders. Their diets include small fish, shellfish, snails, shrimps, crabs, and other benthic organisms (Harmelin‐Vivien et al. 1989; Xue et al. 2017). To obtain potential prey items for isotope baseline comparison, benthic organisms were collected using a Hydrobios Model 50 sediment sampler. The sediments were sieved, and visible benthic fauna were carefully extracted using fine tweezers and transferred into pre‐filtered seawater (filtered through a 0.45 μm membrane) for 24 h to allow gut clearance. These samples were subsequently dried in an oven until they reached constant weight. Additionally, collect shells, snails, shrimps, crabs, and other small fish using gill nets and ground cages.

For stable isotope analysis, collect appropriate amounts of muscle tissue: back muscles from fish, abdominal muscles from shrimps, chelicerae muscles from crabs, adductor muscles from bivalves, and muscle tissue from gastropods. Place all samples in an oven at 60°C and dry them to a constant weight. Then, use a grinder to homogenize the samples into a powder with a uniform particle size. Weigh approximately 250–300 μg of the powdered sample, tightly embed it in a tin cup, and store it dry until analysis.

### 2.3 Stable Isotope Analysis

Stable isotope analysis of all samples was conducted using an Elementar isoprime visION stable isotope ratio mass spectrometer (Elementar, Heraeus, Frankfurt, Germany). The standard material for nitrogen stable isotope analysis is atmospheric nitrogen (N_2_), while the standard for carbon stable isotope analysis is the international standard belemnite (VPDB). In the experiment, one additional standard sample was tested for every 10 samples analyzed, and every 10 samples underwent testing. Additionally, one to two samples from each batch were randomly selected for retesting. The accuracy of the analysis is ±0.3‰. The internationally accepted standard samples include USGS40, USGS41a, and USGS65, among others. The mass spectrometer detects the ^15^N^/14^N ratio in N_2_ and the ^13^C/^12^C ratio in CO_2_ after the reaction, comparing these ratios with the international standard belemnite (VPDB) and atmospheric N_2_ to calculate the stable isotope ratios of the samples. The carbon and nitrogen percentages of the samples are expressed in ‰, while the carbon and nitrogen stable isotope ratios are represented in the form of the internationally accepted *δ* value:

$$\mathrm{\delta X}\left(‰\right)=\left(\frac{R\text{sample}}{R\text{standard}}-1\right)\times 1000$$

In the formula, *X* represents either ^13^C or ^15^N; Rsample denotes the measured isotope ratio, while *R* signifies the ^13^C/^12^C or ^15^N/^14^N ratio.

### 2.4 Data Analysis

Following the method proposed by Layman, a two‐dimensional trophic niche space was constructed using δ^13^C‐δ^15^N biplots. Six quantitative metrics were calculated to assess the trophic niche characteristics of the fish populations (Layman et al. 2007). The δ^15^N range (NR) represents the trophic level span among different fish species, while the δ^13^C range (CR) indicates the breadth of dietary sources. The mean distance to centroid (CD) measures the average trophic diversity, and the total area (TA) of the convex hull represents the total trophic niche space occupied by a species. These four parameters collectively reflect the diversity of population trophic structures. To examine trophic redundancy, two additional metrics were used: the mean nearest neighbor distance (MNND), which characterizes trophic packing density, and the standard deviation of nearest neighbor distance (SDNND), which quantifies the evenness of trophic distribution within the population. The standard ellipse area (SEAc) was used as the primary metric for assessing trophic niche width, as it provides a corrected estimation of core isotopic niche space compared to the total convex hull area (TA). Additionally, the overlap area (OA) between individual SEAc values was defined as an index to quantify trophic niche overlap among individuals (Jackson et al. 2011, 2012). The nicheROVER package in R was used to calculate the 95% Highest Density Interval (HDI) of species' niche breadth. A Bayesian statistical framework was applied to quantify the degree of niche overlap. Standard ellipse areas and niche overlap probabilities were computed within a Bayesian framework using the same package, providing robust measures of core niche width and interspecific dietary convergence. By implementing 10,000 Monte Carlo simulations, this method effectively incorporated uncertainties in the analysis process. The Bayesian mixing model (MixSIAR) was used to estimate the proportional contributions of different prey sources to consumer diets, allowing for probabilistic inference while accounting for uncertainty and isotopic variability. This approach enables probabilistic assessments of trophic niche width and overlap, providing a robust evaluation of resource utilization and interspecific interactions (Swanson et al. 2015). Six potential bait groups were selected: benthic, snails, mollusks, shrimps, crabs, and various small fish. The contribution ratios of these food groups to the diets of *S. marmoratus* and *S. cirrosa* were calculated using the MixSIAR model. This analysis evaluates the dietary composition of both *S. marmoratus* and *S. cirrosa* .

To assess the effects of species and location on the isotopic composition of fish, a two‐way analysis of variance (ANOVA) was conducted separately for δ^13^C and δ^15^N values, with species ( *S. marmoratus* and *S. cirrosa* ) and location (Guishan Island, Wailingding Island, and Dongao & Wanshan Islands) treated as fixed factors. Prior to the analysis, the assumptions of normality and homogeneity of variances were tested using the Shapiro–Wilk and Levene's tests, respectively. A significance threshold was set at *α* = 0.05, and differences were considered statistically significant when *p* < 0.05. Utilizing circular statistics and hypothesis testing, two attributes—length and angle—are employed to represent the directional differences of the two fish species within food webs across various regions (Schmidt et al. 2007). The angle denotes the direction of isotopic niche shifts in δ^13^C–δ^15^N space, corresponding to structural changes in carbon or nitrogen sources and thus indicating shifts in dietary resource types. The length reflects the magnitude of niche displacement, representing the extent of trophic reorganization. This can be interpreted as the strength of a species' response to environmental disturbances or adjustments in foraging strategies. Rayleigh's and Watson–Williams' tests are conducted to assess these differences. If the test results yield significance (*p* < 0.05), it indicates a common movement trend among species and suggests substantial changes in food web structure across time periods or locations. All data analyses and visualizations in this study were conducted using ArcGIS 10.6, Excel, R 4.3.1, SPSS, Origin 2021, and Oriana 4.0.

## 3 Results

### 3.1 Stable Isotopes Signature

The δ^13^C values of *S. marmoratus* ranged from −17.03‰ −14.76‰, with a mean ± standard deviation of −16.01‰ ± 0.60‰. Its δ^15^N values ranged from 11.34‰ to 14.01‰, with a mean of 13.20‰ ± 0.55‰. For *S. cirrosa* , δ^13^C values ranged from −17.89‰ −15.04‰, with a mean of −16.46‰ ± 0.69‰, while δ^15^N values varied between 10.86‰ and 14.11‰, with a mean of 12.47‰ ± 0.79‰. A comparison of the stable isotope values among the three regional groups revealed that *S. marmoratus* from Wailingding Island had the lowest mean δ^13^C value (−16.35‰ ± 0.22‰), whereas *S. cirrosa* from the same island exhibited the highest mean δ^13^C value (−16.80‰ ± 0.56‰). The lowest mean δ^15^N value was observed in *S. cirrosa* from Dongao & Wanshan Islands (12.07‰ ± 0.60‰), while the highest mean δ^15^N value was found in *S. marmoratus* from Wailingding Island (13.32‰ ± 0.25‰).

**FIGURE 2:**
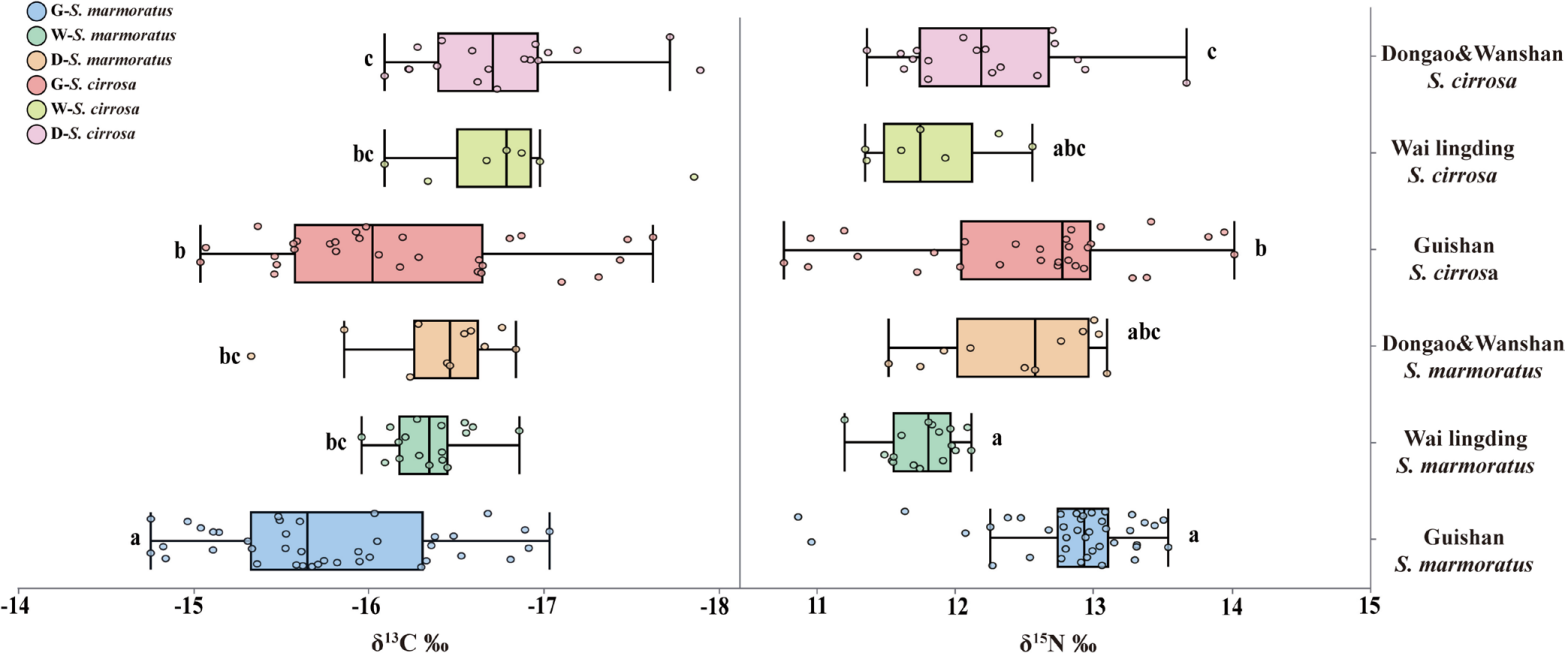
Stable isotope values (δ^13^C and δ^15^N) of *Sebastiscus marmoratus* and *Scorpaenopsis cirrosa* collected from Guishan Island, Wai Lingding Island, and Dongao & Wanshan Island. Different colors represent the two species at each location. Different lowercase letters indicate significant differences among groups (*p* < 0.05).

Two‐way ANOVA revealed significant effects of both species and location on δ^13^C and δ^15^N values, while no significant interaction between the two factors was detected. Specifically, δ^13^C values differed significantly among locations (*F*₂,₁₁₇ = 15.04, *p* < 0.0001) and between species (*F*₁,₁₁₇ = 17.84, *p* < 0.0001), with no interaction effect (*F*₂,₁₁₇ = 0.01, *p* = 0.9900). Similarly, δ^15^N values were significantly influenced by location (*F*₂,₁₁₇ = 8.97, *p* = 0.0002) and species (*F*₁,₁₁₇ = 39.34, *p* < 0.0001), with no significant interaction (*F*₂,₁₁₇ = 0.09, *p* = 0.9176). Post hoc comparisons indicated that, for *S. marmoratus* , both δ^13^C and δ^15^N values at Guishan Island were significantly higher than those observed at Wai Lingding Island and Dongao & Wanshan Islands (*p* < 0.05). For *S. cirrosa* , δ^13^C values were significantly lower at Dongao & Wanshan Islands compared to the other two locations (*p* < 0.05), while δ^15^N values remained relatively consistent across sites. In terms of interspecific comparison within the same location, significant differences in both δ^13^C and δ^15^N were observed at Guishan Island, with *S. marmoratus* exhibiting higher isotopic values than *S. cirrosa* (*p* < 0.05). At the other two locations, the isotopic values between the two species did not differ significantly. These patterns are visually represented in the boxplots (Figure 2).

### 3.2 Trophic Structure of Fish Communities Across Different Island Regions

**FIGURE 3:**
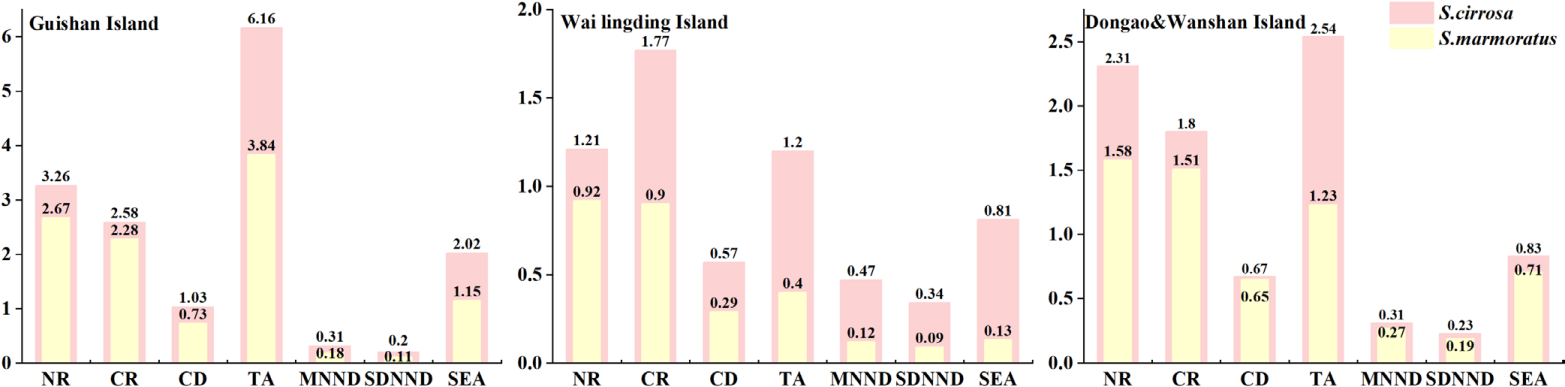
Trophic niche structure indices of *Sebastiscus marmoratus* and *Scorpaenopsis cirrosa* at Guishan Island, Wai Lingding Island, and Dongao & Wanshan Island. Different bars represent values for the two species at each location. CD, centroid distance; CR, carbon range; MNND, mean nearest neighbor distance; NR, nitrogen range; SDNND, standard deviation of nearest neighbor distance; SEA, standard ellipse area; TA, total area of convex hull.

The trophic niche indicators of *S. marmoratus* and *S. cirrosa* vary across different regions (Figure 3), highlighting the distinct food web structures present in these areas. In the Guishan Island sea area, the trophic niche indicators for both *S. marmoratus* and *S. cirrosa* are higher compared to those observed at Wai Lingding Island and Dongao & Wanshan Island, with Wai Lingding Island exhibiting the lowest trophic niche indicators. Furthermore, the trophic niche indicators across the three groups of islands consistently show that *S. cirrosa* possesses higher values than *S. marmoratus* . This suggests that *S. cirrosa* has access to a broader range of food sources and occupies a more extensive trophic niche space, whereas *S. marmoratus* demonstrates greater nutritional redundancy and a more uniform distribution of trophic niches.

**FIGURE 4:**
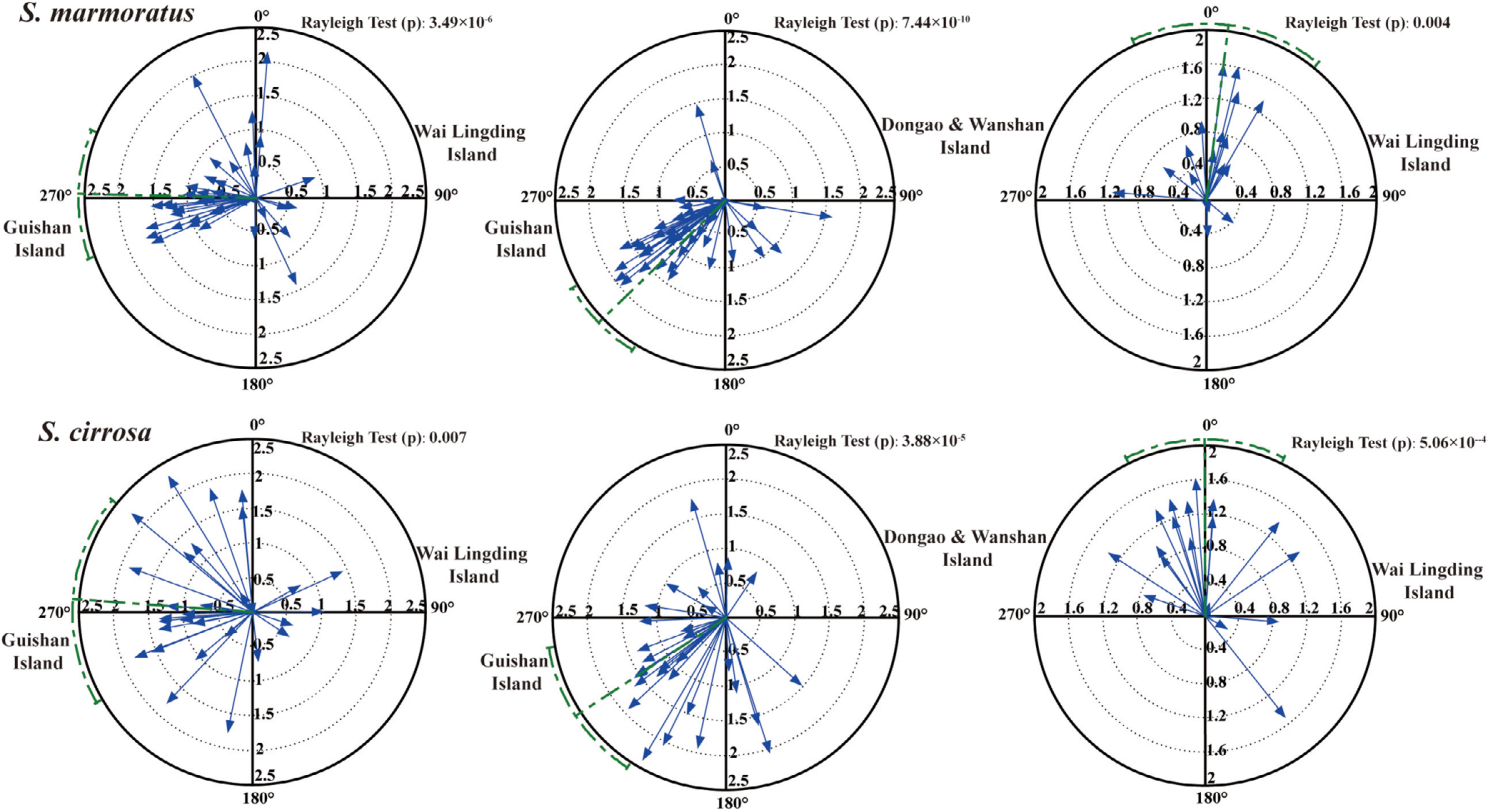
Directional changes in the trophic structure of *S. marmoratus* and *S. cirrosa* at varying islands.

The trophic structure of *S. marmoratus* and *S. cirrosa* across different island habitats exhibited distinct directional changes in isotopic niche space, as shown by the circular statistical vector diagrams (Figure 4). In this context, the angle of change reflects the primary direction of trophic shift, while the magnitude represents the degree of variation in trophic niche characteristics. When comparing Guishan Island with Wai Lingding Island and with Dongao & Wanshan Islands, both species showed consistent directional changes with large vector magnitudes, indicating notable spatial differences in trophic niche configuration. In contrast, between Wai Lingding Island and Dongao & Wanshan Islands, the directional shifts were smaller and vector lengths shorter, suggesting only minor trophic adjustments.

### 3.3 Isotopic Niche Analysis

**FIGURE 5:**
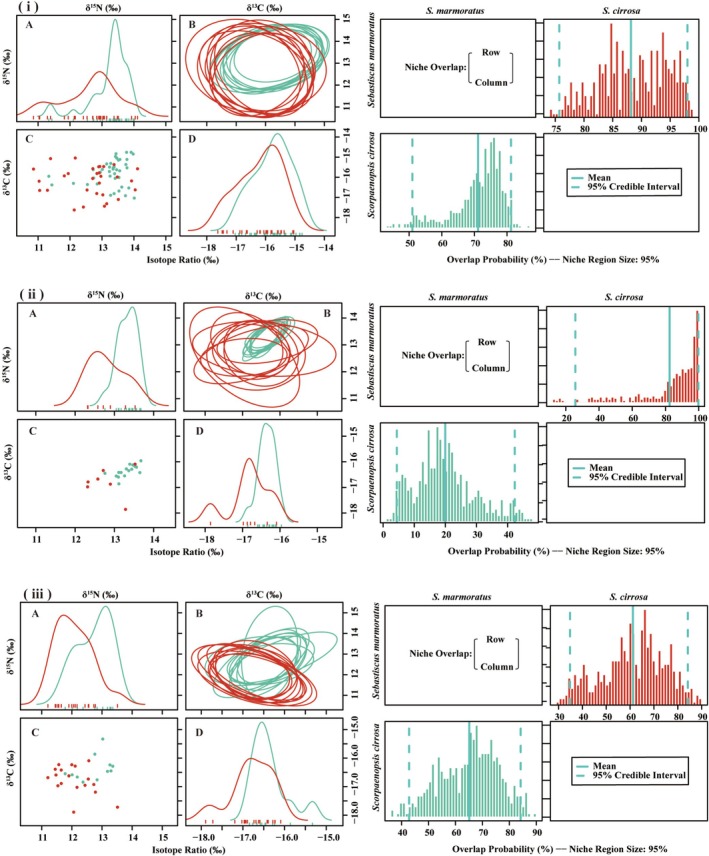
Left: 10 random elliptical projections of niche region for two investigated fish species ( *S. marmoratus* and *S. cirrosa* ) across three island groups (i: Guishan Island, ii: Wai Lingding Island, iii: Dongao & Wanshan Island), including smoothed histograms (A and D) and scatterplot (C). Red and green represent *S. marmoratus* and *S. cirrosa* , respectively. Right: Posterior distribution of niche overlap metric (%) for a specified niche range of 95% across the three island groups. The posterior means and 95% credible interval are displayed in light green.

The δ^13^C and δ^15^N isotopic distributions of *S. marmoratus* and *S. cirrosa* exhibited significant regional variation across Guishan Island, Wailingding Island, and Dongao & Wanshan Islands (Figure 5). Overall, *S. marmoratus* displayed a broader range of δ^13^C and δ^15^N values across all islands, whereas *S. cirrosa* showed a more constrained isotopic distribution, particularly with lower variability in carbon isotope ratios (Figure 5A,D). The projection of 95% confidence ellipses revealed varying degrees of trophic niche overlap between *S. marmoratus* and *S. cirrosa* among the islands. At Guishan Island, the two species exhibited a higher degree of niche overlap, suggesting potential resource sharing in this region (Figure 5B). In contrast, at Wailingding Island and Dongao & Wanshan Islands, the niche overlap was more limited, indicating a greater degree of resource partitioning and a more distinct niche separation pattern. The posterior overlap probability distribution further illustrated the differences in niche overlap across islands. In all regions, *S. cirrosa* was generally more likely to fall within the trophic niche of *S. marmoratus* , but the extent of overlap varied among islands.

### 3.4 Food Source Analysis

**FIGURE 6:**
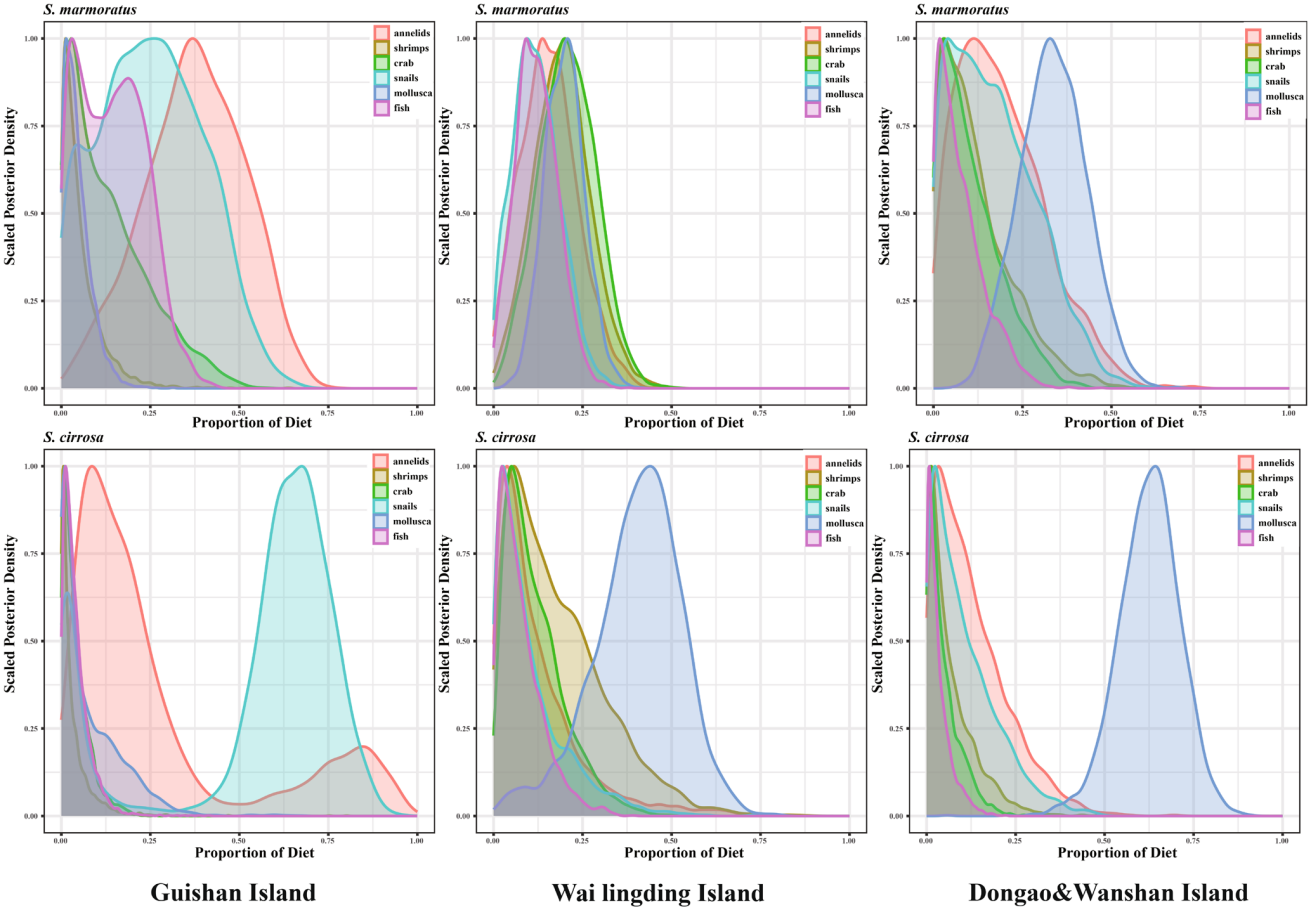
Posterior density distribution of dietary proportions for *S. marmoratus* and *S. cirrosa* at Guishan Island, Wai Lingding Island, and Dongao & Wanshan Islands.

**FIGURE 7:**
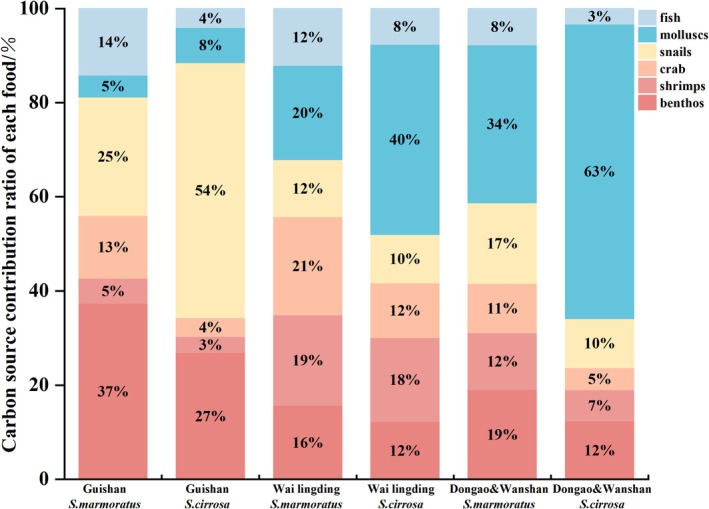
Contribution ratio of carbon sources to food of *S. marmoratus* and *S. cirrosa* in Guishan Island, Wai lingding Island, and Dongao&Wanshan Island.

The Bayesian mixing model produced probability density distribution curves for the contribution proportions of six potential prey groups to the diets of *S. marmoratus* and *S. cirrosa* (Figure 6), as well as the percentage contributions of these prey groups across different regions (Figure 7). At Guishan Island, annelids and snails were the dominant dietary components for both species. *S. marmoratus* showed a higher reliance on annelids, which contributed 37% to its diet, whereas *S. cirrosa* exhibited a greater dependence on snails, with a contribution rate of 54%. In contrast, at Wailingding Island and Dongao & Wanshan Islands, the consumption of annelids by both species was significantly lower than at Guishan Island. Notably, at Wailingding Island, *S. cirrosa* displayed an increased consumption of crabs and mollusks. A particularly strong preference for mollusks was observed in *S. cirrosa* , especially at Dongao & Wanshan Islands, where mollusks accounted for 63% of its diet—substantially higher than any other prey group. Additionally, *S. marmoratus* consistently consumed small benthic fishes at higher proportions than *S. cirrosa* across all three island groups. At Wailingding Island, both species exhibited a more diversified feeding pattern, suggesting a more diversified dietary composition.

## 4 Discussion

### 4.1 Disparities in Stable Isotope Ratios

Due to the close resemblance of organisms' carbon isotopes to those of their food sources, carbon isotopes are predominantly utilized in food source analyses, with δ^13^C values serving as a critical indicator in aquatic ecosystems (Perkins et al. 2014). In contrast, nitrogen isotopes become progressively enriched along the food chain, rendering δ^15^N values a reliable metric for assessing trophic levels (Jennings and Cogan 2015). In this study, the δ^15^N values of *S. marmoratus* and *S. cirrosa* exhibited no inter‐regional differences; however, the δ^13^C values demonstrated significant variations exclusively in the Guishan Island region, indicating distinct carbon sources in that area compared to others. These discrepancies suggest considerable variation in habitat conditions and food sources across regions (Perkins et al. 2014; Camilleri and Ozersky 2019). Regional differences in benthic primary productivity and variations in organic carbon sources across water bodies contribute to the diverse δ^13^C values observed among geographic locations (Riva‐Murray et al. 2013). Although both species are benthic‐feeding, low‐trophic‐level carnivores (Xue et al. 2017) environmental conditions and food availability have resulted in differing feeding preferences (Koussoroplis et al. 2010). Similar observations were noted in *Gymnocephalus cernua* across two Czech lakes, where habitat differences influenced resource dependence and competition levels, thereby affecting fish diets (Eloranta et al. 2017). Furthermore, a study on spatial variation in δ^15^N across the northeastern Atlantic Ocean underscored the impact of regional environmental factors on δ^15^N at the base of the food chain, which in turn affects the δ^15^N values of *Limanda limanda* and *Merlangius merlangus* (Jennings and Warr 2003). However, while δ^15^N values are influenced by environmental nitrogen sources, they also reflect a species' position within the food chain (Post 2002). Consequently, the similar trophic levels of *S. marmoratus* and *S. cirrosa* result in closely matched δ^15^N values, indicating no significant inter‐regional differences. A comparison of δ^13^C and δ^15^N values between the two species revealed distinct differences in the Guishan Island region, highlighting their varied feeding preferences in relation to their positions in the food chain (Perkins et al. 2014; Jennings and Cogan 2015).

Quantitative analyses of stable isotope values in *S. marmoratus* and *S. cirrosa* across various regions revealed subtle differences among three groups, with consistent directional variations in δ^13^C and δ^15^N. These findings suggest that the environmental or resource conditions between Wai Lingding Island and the Dongao & Wanshan Islands are similar, while the Guishan Island area differs significantly in terms of trophic niche structure and food composition, indicating a more complex food web structure (Schmidt et al. 2007). The distinct characteristics of Guishan Island may be attributed to its higher human population and greater levels of human disturbance, which likely influence fish feeding strategies. Guishan Island is geographically the closest to the mainland and is served by the most frequent ferry routes departing from Xiangzhou Port in Zhuhai City, which is the only mainland port connecting to these nearshore islands. Based on our compilation of ferry schedules (11 trips/day to Guishan Island, 8 to Wai Lingding Island, 6 to Dongao Island, and 3 to Wanshan Island), Guishan Island receives significantly more daily ferry services than Wai Lingding Island and Dongao & Wanshan Islands, indicating higher levels of human interaction and accessibility. Moreover, anthropogenic impacts on estuarine and coastal environments generally diminish with increasing distance from the mainland (Lotze et al. 2006). Therefore, Guishan Island is likely subject to stronger and more sustained human influences, which may contribute to the observed variations in local food web dynamics and trophic structure. For instance, a study on land use changes and trophic organization in fish within stream systems observed significant dietary differences between undisturbed and human‐disturbed mangrove streams, as indicated by δ^13^C and δ^15^N values in fish muscle (Mwandya 2019). Additionally, research on isotopic trophic niches of fish in a heavily disturbed river in tropical China found a gradient in δ^13^C and δ^15^N values from upstream to downstream, with a sharp decrease observed in the industrial area (Wang et al. 2021). Anthropogenic nutrient inputs, particularly from nitrogen sources, can alter baseline isotope values and influence trophic behavior among fish species. Such inputs, along with habitat degradation, may shift δ^13^C baselines and compress food web structures, thereby reducing trophic space and constraining resource partitioning (De Carvalho et al. 2021).

### 4.2 Analysis of Trophic Niche Structure and Geographical Variability

This study analyzed the variation in CR and NR, mean centrifugal distance (CD), and total area of trophic niche (TA) for *S. marmoratus* and *S. cirrosa* . The results indicated that *S. cirrosa* utilizes a broader range of food sources and occupies a more extensive range of trophic niches both regionally and overall. A significant positive correlation was observed between the strength of environmental tolerance, trophic niche width, habitat width, and feeding width (Slatyer et al. 2013). Additionally, most fish are selective foragers, choosing the type and size of food based on the available resources in their environment (Turesson et al. 2002). For example, *Chaetodon triangulum* consumes fewer coral species in resource‐rich environments compared to resource‐poor areas. This difference arises because, in conditions of scarce resources, organisms broaden their diet to sustain growth, whereas in abundant conditions, they select food based on preference (Chandler et al. 2016). These findings suggest that *S. cirrosa* is more adaptable to varying habitats and utilizes a wider array of food resources than *S. marmoratus* . Furthermore, *S. cirrosa* exhibits a higher population trophic aggregation density (MNND) across all regions, indicating more extensive feeding, lower trophic redundancy, and more efficient resource utilization, which are indicative of trophic niche differentiation (Layman et al. 2007).

Species population sizes, food resource density and diversity, as well as competitive and predatory interactions, collectively shape the trophic niches of species (Bearhop et al. 2004). The overlap of trophic niches reflects the degree to which different species utilize the same resources, serving as a key indicator of competition intensity between species (Dehnhard et al. 2020; Pastore et al. 2021). In this study, we observed that *S. marmoratus* and *S. cirrosa* in the waters of Guishan Island exhibited the widest trophic niche breadth among the three regions and demonstrated significant trophic niche overlap. This high degree of resource overlap may be attributed to the greater human population and higher levels of anthropogenic disturbance on Guishan Island, which resulted in reduced bait diversity and more limited resources, consequently increasing trophic niche overlap between the two species (Smith et al. 2018). The increased niche similarity under such conditions may signal heightened interspecific competition, as has also been observed in other disturbed ecosystems where human activities compress available trophic space and restrict resource specialization (Olds et al. 2014). A similar study conducted in the sub‐shallow waters of Erhai Lake, China, found that two goby species of the same genus ( *Rhinogobius cliffordpopei* and *Rhinogobius giurinus* ) exhibited significant trophic niche overlap; however, in deeper waters, they employed different strategies such as spatial segregation and trophic niche differentiation. This suggests that fish interactions may be influenced by environmental factors, including habitat type and food resource availability (Guo et al. 2015). Furthermore, the trophic niches of *S. marmoratus* and *S. cirrosa* were narrower and showed some degree of differentiation on Wai Lingding Island and Dongao & Wanshan Island, with more pronounced differentiation on Wai Lingding Island. This likely reflects differences in habitat types, environmental conditions, and food resources across the islands, driving changes in the dietary and behavioral adaptations of the fish (Marklund et al. 2017). These differences are critical in shaping species' feeding strategies and driving trophic niche differentiation (Darimont et al. 2009). For instance, a long‐term study of the feeding habits of *Gymnogobius isaza* in Lake Biwa, Japan, found that shifts in environmental conditions and food availability led to a change in its primary food source from zooplankton (e.g., copepods and branchiopods) to benthic fauna (e.g., gammarids), significantly altering its feeding preferences (Briones et al. 2012). This underscores how anthropogenic disturbances and environmental factors can influence fish feeding behavior. The study by Marklund et al. (2017) also demonstrated that habitat accessibility affects feeding specialization and trophic niche utilization in * *Perca fluviatilis* *. It was shown that increased habitat conversion leads to reduced specialization in feeding preferences and niche use. Conversely, minimal habitat conversion supports higher feeding preferences and greater trophic niche utilization (Marklund et al. 2017). This finding further suggests that Wai Lingding Island and Dongao & Wanshan Island, which experience less anthropogenic disturbance, enable *S. marmoratus* and *S. cirrosa* to mitigate competition by adjusting or specializing their feeding strategies, thereby promoting trophic niche differentiation (Slatyer et al. 2013). This result is consistent with findings from the temperate east coast of Australia, where no significant food overlap was observed across a latitudinal gradient, even among fish populations with similar foraging types (Kingsbury et al. 2020). Additionally, favorable environmental conditions and abundant food resources in the Coaracy Nunes Reservoir in the Amazon allow fish species to select food sources based on specific preferences, reducing competition and facilitating species distribution according to their trophic niche needs, thereby minimizing trophic niche overlap (Sa‐Oliveira et al. 2014). Thus, the diversity of environmental factors and food resources on Wai Lingding Island and Dongao & Wanshan Island provided a wider range of feeding options than on Guishan Island, contributing to the trophic niche differentiation observed between *S. marmoratus* and *S. cirrosa* .

### 4.3 Impact of Geographical Differences on Food Sources and Ecological Strategies

Geographical variation significantly influences the availability of food resources, thereby shaping the ecological strategies of fish species (Alves et al. 2021). Differences in habitat and species competition can alter the feeding preferences of fishery species, while shifts in food sources directly affect the carbon and nitrogen stable isotope signatures within organisms (Jennings and Cogan 2015; Alves et al. 2021). Food source analysis serves as a fundamental approach for studying the trophic niche of aquatic organisms, as interspecific differences in dietary choices and interactions can influence niche width (Chang et al. 2014; Segura‐Cobena et al. 2021). In this study, *S. marmoratus* and *S. cirrosa* exhibited significant differences in dietary composition across islands. Both species consumed considerably less benthos at Wailingding Island and Dongao & Wanshan Islands compared to Guishan Island, while their consumption of bivalves increased significantly. This pattern suggests that food resources were relatively more abundant at Wailingding Island and Dongao & Wanshan Islands, leading to a stronger preference and selectivity for bivalves (Winemiller et al. 2001). Studies indicate that while water environmental factors do not directly affect trophic niches, they indirectly influence fish dietary composition by regulating the abundance and distribution of food resources (Sha et al. 2015). For example, a study on food web dynamics in two major estuaries along the Portuguese coast found that trophic interactions varied both seasonally and spatially, with spatial differences in fish isotopic composition (among different estuarine regions) being more pronounced than seasonal variations (across different months) (França et al. 2011). Furthermore, human activities such as coastal development and fishing can alter food web structures and the distribution of food resources, prompting species to modify their dietary sources or develop new ecological strategies to adapt to habitat changes (Doherty et al. 2021). Guishan Island experiences higher levels of human disturbance, and both shrimps and bivalves are important fishery targets. As a result, differences in food availability directly influence the feeding behavior and trophic strategies of fish (Pratiwy et al. 2023). When food resources are scarce, fish may broaden their prey selection criteria, opting for food sources that offer lower energy yields but are more readily available (Killen 2011). Conversely, when food resources are abundant, feeding preferences and behaviors are more influenced by individual assessments of food quality (Hansen et al. 2016). This pattern was also observed in the present study. At Wailingding Island, both *S. marmoratus* and *S. cirrosa* exhibited relatively balanced dietary choices. However, compared to Guishan Island, *S. marmoratus* showed a significantly higher consumption of shrimps, displaying a clear preference for them, while *S. cirrosa* exhibited a strong preference for mollusks. Nahon et al. analyzed fish feeding preferences and behaviors using fatty acids (FA) and carbon and nitrogen stable isotopes (δ^13^C and δ^15^N). Their study indicated that when food resources are abundant, fish feeding preferences are primarily driven by the high energy and nutritional value of prey, although they still adjust their diet based on the availability of food resources in their environment. In areas where food resources are relatively scarce, significant dietary overlap occurs among fish species. Additionally, fish exhibit feeding flexibility to ensure adequate energy intake, even when the energy returns from available prey are relatively low (Nahon et al. 2024). Studies on food specialization in juvenile fish communities during the summer have shown that fish tend to develop dietary specialization during periods of high seasonal resource abundance. This adaptation allows them to take advantage of plentiful food supplies, coexist in diverse environments, and achieve rapid growth (Keast 1985). Resource‐rich environments provide fish communities with a diverse range of food sources and niche opportunities, enabling species to specialize in their feeding habits by focusing on specific resources. This reduces competition and facilitates species coexistence (Then and Chong 2022). Research on potential trophic competition among benthic fish in different substrate types has indicated that in resource‐limited environments, fish broaden their resource use to expand their trophic niche width. This strategy mitigates competitive pressures by increasing dietary flexibility (Pelage et al. 2022). These findings align with the trophic niche characteristics of *S. marmoratus* and *S. cirrosa* observed across different islands. Furthermore, the greater dietary selectivity observed at Wailingding and Dongao & Wanshan Islands likely reflects relatively higher habitat quality and prey availability. Such conditions buffer species from trophic niche overlap and facilitate more stable interspecific coexistence. Protecting structurally complex and less‐disturbed habitats is therefore essential for maintaining trophic diversity and ecological resilience in reef‐associated fish communities.

## 5 Conclusion

This study utilized stable isotope analysis to investigate the trophic niche characteristics and geographical variations of *S. marmoratus* and *S. cirrosa* in the Wanshan Archipelago. The findings revealed significant differences in trophic niche width and overlap between the two species across different islands. The δ^13^C and δ^15^N values of *S. marmoratus* and *S. cirrosa* provided preliminary insights into habitat variations among islands and the varying degrees of human disturbance. Bayesian mixing model analysis further elucidated differences in dietary contributions across regions, reflecting distinct feeding strategies and a certain degree of trophic niche differentiation. The higher level of human disturbance at Guishan Island resulted in a relatively lower availability of food resources compared to the other two island groups. Consequently, *S. marmoratus* and *S. cirrosa* exhibited broader trophic niches and wider dietary breadths to meet their energy requirements. However, at Wailingding Island and Dongao & Wanshan Islands, both species displayed distinct feeding preferences. The broader isotopic niche of *S. cirrosa* suggests greater adaptability and a wider resource utilization range. Meanwhile, the trophic niche overlap between the two species indicates potential competition for food resources.

## Author Contributions

**Hongyu Xie:** conceptualization (equal), data curation (equal), methodology (equal), software (equal), visualization (equal), writing – original draft (lead). **Yu Liu:** investigation (equal), methodology (equal), validation (equal), visualization (equal), writing – review and editing (equal). **Teng Wang:** data curation (equal), investigation (equal), methodology (equal), validation (equal), visualization (equal), writing – review and editing (equal). **Peng Wu:** conceptualization (equal), formal analysis (equal), project administration (equal). **Yayuan Xiao:** conceptualization (equal), formal analysis (equal), project administration (equal). **Chunling Wang:** conceptualization (equal), formal analysis (equal), project administration (equal). **Jian Zou:** conceptualization (equal), formal analysis (equal), project administration (equal). **Yong Liu:** formal analysis (equal), funding acquisition (equal), investigation (equal), project administration (equal), writing – review and editing (equal). **Jinhui Sun:** conceptualization (equal), methodology (equal), supervision (equal). **Jianzhong Shen:** conceptualization (equal), methodology (equal), supervision (equal). **Xuefu Ao:** conceptualization (equal), methodology (equal), supervision (equal). **Yanqiao Wang:** conceptualization (equal), data curation (equal), formal analysis (equal), validation (equal).

## Ethics Statement

All field sampling was conducted in accordance with local fisheries regulations and institutional guidelines. No endangered or protected species were involved in this study. All procedures involving animals were conducted under the supervision of the South China Sea Fisheries Research Institute, Chinese Academy of Fishery Sciences, and were approved by its institutional ethics committee. The ethical review number is nhdf2025‐17.

## Conflicts of Interest

The authors declare no conflicts of interest.

## Data Availability

The data that support the findings of this study are openly available in [Dryad] at https://doi.org/10.5061/dryad.v15dv427h.
