# Correction to “The Influence of Habitat Heterogeneity and Human Disturbance on Trophic Interactions of *Sebastiscus marmoratus* and *Scorpaenopsis cirrosa* in Wanshan Archipelago”

**DOI:** 10.1002/ece3.72103

**Published:** 2025-09-10

Xie, H., Y. Liu, T. Wang, et al. 2025. “The Influence of Habitat Heterogeneity and Human Disturbance on Trophic Interactions of *Sebastiscus marmoratus and Scorpaenopsis cirrosa* in Wanshan Archipelago.” *Ecology and Evolution* 15, no. 7: e71869. https://doi.org/10.1002/ece3.71869.

In paragraph 1 of the “Funding” section, the text “This work was supported by Financial Fund of the Ministry of Agriculture and Rural Affairs, P. R. China (NHZX2024), Science and Technology Planning Project of Guangdong Province (2019B121201001), Central Public‐interest Scientific Institution Basal Research Fund, CAFS (2023TD16, 2024RC03), National Key R&D Program of China (2024YFD2401801), South China Sea Fisheries Research Institute, CAFS (2022TS01, 2024RC13, 2025RC03).” was incorrect.

This should have read: “This work was supported by National Key R&D Program of China (2024YFD2401801), Central Public‐interest Scientific Institution Basal Research Fund, South China Sea Fisheries Research Institute, CAFS (2022TS01, 2025RC03, 2024RC13), Financial Fund of the Ministry of Agriculture and Rural Affairs, P. R. China (NHZX2024), Central Public‐interest Scientific Institution Basal Research Fund, CAFS (2023TD16, 2024RC03), Science and Technology Planning Project of Guangdong Province (2019B121201001).”

We apologize for this error.

